# Cost-effectiveness of 20-valent pneumococcal conjugate vaccine compared with 23-valent pneumococcal polysaccharide vaccine among adults in a Norwegian setting

**DOI:** 10.1186/s12962-023-00458-4

**Published:** 2023-08-09

**Authors:** Mikkelsen Malene B, Husby Oyvind, Molden Tor, Mwaura David N, Olsen Jens, Kristensen Nanna V, Vietri Jeffrey

**Affiliations:** 1 EY, Copenhagen, Denmark; 2 Pfizer Norway, Drammensveien 288, Oslo, 0283 Norway; 3 Pfizer Inc, Collegeville, PA USA

**Keywords:** Pneumococcal diseases, Pneumococcal infection, Vaccination, Cost-utility analysis, PCV20, 20-valent pneumococcal conjugate vaccine, Cost-effectiveness, Pneumonia

## Abstract

**Background:**

The morbidity and mortality of adult diseases caused by *S. pneumoniae* increase with age and presence of underlying chronic diseases. Currently, two vaccine technologies against *S. pneumoniae* are used: the 23-valent pneumococcal polysaccharide vaccine (PPV23) and the pneumococcal conjugate vaccines, one of which is the 20-valent pneumococcal conjugate vaccine (PCV20) that has recently been approved for adults.

**Objective:**

This study was conducted to investigate the cost-effectiveness of implementing PCV20 in a reimbursement scheme for Norwegian adults aged 18–99 years at risk of pneumococcal diseases and those aged 65 years and older at low risk compared to PPV23.

**Methods:**

An established Markov model was adapted to a Norwegian setting to estimate the economic and clinical consequences of vaccinating the Norwegian population in specific age and risk groups against pneumococcal diseases. Inputs for the model were found in Norwegian or Danish real-world evidence or retrieved from available studies. The costs and clinical outcomes were assessed using a health sector perspective and a lifetime time horizon.

**Results:**

The results showed that PCV20 was associated with better health outcomes including fewer disease cases, fewer disease-attributable fatalities, a higher gain of life years and quality-adjusted life years compared to PPV23. In addition, PCV20 had a lower total cost compared to PPV23. Therefore, PCV20 was the dominant vaccination strategy. The base case result was investigated in multiple sensitivity analyses, which showed that the results were robust to changes in input parameters and methodological assumptions, as PCV20 remained the dominant vaccination strategy in almost all scenarios.

**Conclusion:**

Results showed that vaccinating the Norwegian adults with PCV20 was cost-effective compared to PPV23. Changes in the hospital cost of pneumonia, the price of PCV 20, the effectiveness of PCV20 against pneumonia, and the pneumonia disease incidence had the highest impact on the ICER, i.e., were the main drivers of the results.

## Introduction

Pneumococcal diseases are common infections caused by the bacterial species *S. pneumoniae*. Worldwide, it is an important cause of infection and death among both children and adults [1, 2]. Infections with *S. pneumoniae* include both invasive pneumococcal diseases (IPD), described as meningitis, bacteraemia, and bacterial pneumonia, and non-invasive pneumococcal diseases, such as community-acquired pneumonia (CAP) [1–3]. In Europe, *S. pneumoniae* is responsible for 20–30% of all CAP cases, and it is well known that the non-invasive pneumococcal diseases are three times more frequent than IPD in hospitalised adults [4, 5]. The pathogenicity and invasiveness of *S. pneumoniae* is determined by the composition of the polysaccharides in the capsule, which define the serotypes of the bacteria; currently, 100 distinct serotypes of *S. pneumoniae* are known [2, 6].

The morbidity and mortality of pneumococcal diseases increase with age and the presence of underlying chronic diseases [4, 7, 8]. To prevent IPD and CAP, vaccines have been developed, and currently two types of pneumococcal vaccines are available, the 23-valent pneumococcal polysaccharide vaccine (PPV23) and pneumococcal conjugate vaccines (PCVs). The vaccines are based on different technologies and thus induce different immune responses [9]. Vaccination with PCVs provide a robust T-cell dependent immunisation as well as immunological and mucosal memory, which is not induced by PPV23 [10, 11]. In addition, vaccination with PCVs provide longer-lasting effects than PPV23 [9, 12]. In a recent review of studies, the effectiveness of the 13-valent pneumococcal conjugate vaccine (PCV13) and PPV23 was investigated on the same outcomes using similar methods and populations and found the conjugate vaccine to provide a superior protection against both pneumococcal disease and respiratory infections more broadly [13]. But on the other hand PCVs in Norway are associated with a higher price than PPV23 [14].

In Norway, vaccination against pneumococcal diseases is primarily financed in the childhood vaccination programme, in which the 7-valent pneumococcal conjugate vaccine (PCV7) was introduced in 2006 and replaced by PCV13 in 2011 [5]. Introduction of PCVs in the childhood vaccination programme has resulted in a decrease of the incidence of IPD caused by *S. pneumoniae* serotypes covered by PCV13 in all age groups [15]. In addition, PCV13, as well as the recently-approved 15- and 20-valent pneumococcal conjugate vaccines (PCV15 and PCV20), are currently financed for selected medical high-risk groups and given in series with PPV23 according to the blue prescription (“blå resept”) for people who are stem cell-transplanted or HIV-positive and people with functional or anatomic asplenia [5, 16]. PCV13 and PCV15 are approved for both children aged six weeks to 17 years and adults, whereas PCV20 is only approved for adults. Even though pneumococcal vaccination is not financed in any other risk groups in Norway, it is currently recommended that people with increased risk of IPD receive PPV23, as it covers more serotypes than PCV13, PCV15 and PCV20.

However, the broader serotype protection of PCV15 and PCV20 compared to PCV13 narrows the serotype coverage gap between PCVs and PPV23 [11]. In 2020 and 2021, 53% and 65% of the reported cases of IPD in Norway were caused by serotypes covered by PCV20 and PPV23, respectively [11]. The protection against CAP and IPD in PCV13 has been demonstrated in the Community-Acquired Pneumonia Immunization Trial in Adults (CAPITA) study [17]. As the serotypes of PCV13 are all included in PCV20, and PCV20 has shown non-inferiority to PCV13, similar effects against CAP and IPD can be expected. In contrast, studies have shown inconclusive results regarding the vaccine efficacy of PPV23 against CAP [13, 18, 19].

Based on the burden of pneumococcal diseases and the economic impact of the diseases, this study aimed to investigate the cost-effectiveness of PCV20 vaccination of Norwegian adults aged ≥ 18 years at risk of pneumococcal diseases and all adults aged ≥ 65 years at low and at risk of pneumococcal diseases compared to PPV23. In the model, the at-risk group includes both immunocompetent adults (typically considered at moderate risk of pneumococcal diseases) and adults with immunocompromising conditions, who are not part of the current blue prescription scheme (typically considered at high risk of pneumococcal diseases).

## Methods

**Fig. 1:**
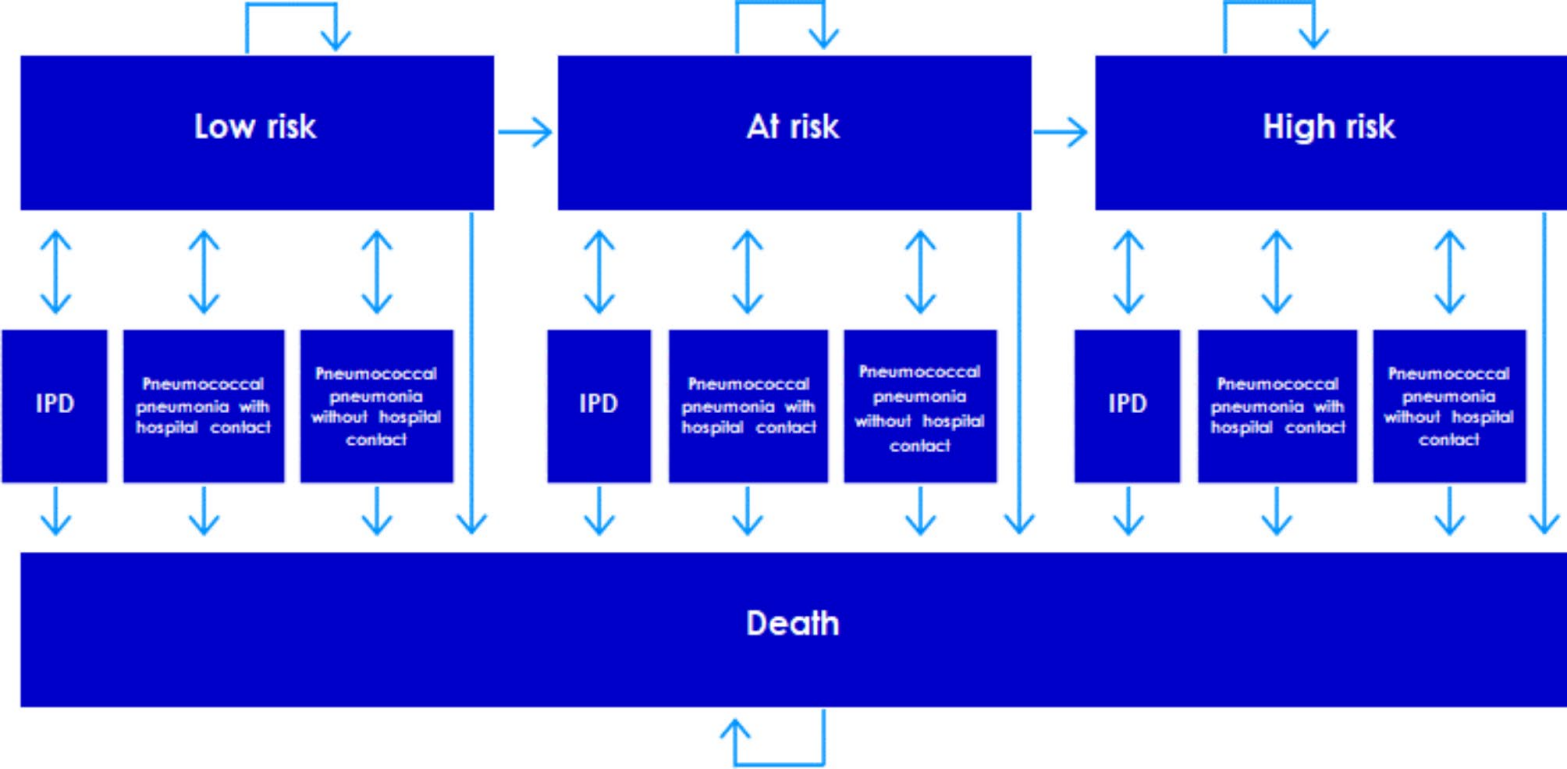
Model structure

To investigate the cost-effectiveness of PCV20 for adults in Norway, a cost-utility model was adapted to a Norwegian setting. The model has previously been adapted to a Danish setting and is also described in a study by Olsen et al. [20]. The cost-utility analysis was conducted using a Markov transition model with one-year cycles. In the model, the possible transitions of each cycle are related to patients experiencing an event of IPD, defined as meningitis or bacteraemia in the model, or pneumonia with or without hospital contact. When patients experience a pneumococcal disease, they can either die or recover. In addition, patients can experience all three types of pneumococcal disease within one cycle. In Fig. 1, an overview of the model structure and the possible transitions are provided.

The study population was stratified by age and risk of pneumococcal diseases. The age groups included in the model were adults aged 18–49 years, 50–64 years, 65–74 years, 75–84 years, and 85–99 years. Based on 2019 data from Statistics Norway, the number of people in each age group was estimated. 2019 data was used because it represents the most recent data not influenced by the COVID-19 pandemic [21]. Due to the initiatives introduced during the COVID-19 pandemic, such as lockdowns and social distancing, fewer cases of pneumococcal diseases were observed; however, it is expected that the number of disease cases will return to their pre-pandemic levels. This is already apparent in Norwegian IPD data, which show that the total number of IPD cases in 2018 and 2019 was 582 and 600 cases, during the pandemic in 2020 and 2021 the number of IPD cases was 294 and 318, respectively. In 2022, the total number of IPD cases was 517 and thus, almost returned to the level seen in 2018 and 2019 [22]. In each age group, the population was stratified to be at low risk, at risk or high risk of pneumococcal disease. The share of people in each age and risk group were determined using the 10th edition of the International Classification of Diseases (ICD-10) codes to identify at-risk and high-risk diseases, and the share of patients in each risk group was determined using data for anyone with a diagnosis corresponding to the risk groups between 2015 and 2019 from the Norwegian Patient Registry (NPR) [23]. The at-risk group is, as stated, a combination of patients at both moderate and high risk of pneumococcal disease for whom the pneumococcal vaccines are currently not reimbursed. Therefore, people at low risk constituted the remaining share of the population in each age group. The share included in each age and risk group is provided in Table 1. In the model, it is possible for the population to change risk group to a higher level of risk (see Fig. 1). A closed cohort was used and therefore the model did not include a new generation in each cycle.

**Table 1:** Number of people in each age group and their distribution into risk groups

	Number of people	Percentage of people in each age and risk group		
Age		Low risk	At risk	High risk
18–49 years	2,291,376	80.10%	19.71%	0.19%
50–64 years	995,487	59.86%	39.93%	0.21%
65–74 years	528,795	42.16%	57.73%	0.11%
75–84 years	273,257	30.05%	69.87%	0.08%
85–99 years	115,710	37.45%	62.50%	0.05%

Note: Risk groups refer to an individual’s risk of pneumococcal diseases based on the presence of underlying chronic conditions. Low risk refers to adults with no underlying chronic conditions. At risk refers to immunocompetent adults with underlying chronic condition and immunocompromised adults, who are not currently included in the Norwegian blue prescription (“blå resept”). High risk refers to immunocompromised adults who are currently included in the blue prescription. Sources: [21, 23]

The model included a lifetime time horizon to capture costs and effects of the different vaccination strategies. The time horizon is estimated based on the model having an upper age limit of 99 years. As the model used a closed cohort, the length of the lifetime time horizon is restricted to the age of the cohort at cycle 0 implying a time horizon of 81 years (99 years minus 18 years).

Based on Norwegian guidelines, the included discount rate was 4% in model years 0–39, 3% in model years 40–74, and 2% from model year 75 onwards [24]. The perspective included in the model was a healthcare sector perspective, meaning that only costs accrued by the public healthcare sector were included.

### Incidence and mortality of IPD and pneumonia

The disease incidences for meningitis and pneumonia with or without hospital contact are based on data from NPR and the Norwegian Registry for Primary Health Care and were estimated per 100,000 [23, 25]. Based on the data available the incidence of bacteraemia was calculated as the incidence of meningitis subtracted from the incidence of IPD. The incidence of IPD cases was identified through the Norwegian surveillance system for communicable diseases registry [22]. Inputs regarding the incidence are presented in Table 2.

**Table 2:** IPD and pneumonia incidence and mortality according to age and risk group

		Incidence				Mortality				
Age group	**Risk group**	**Bacteraemia**	**Meningitis**	**Pneumonia with hospital contact**	**Pneumonia without hospital contact**	**General population**	**Bacteraemia**	**Meningitis**	**Pneumonia with hospital contact**	**Pneumonia without hospital contact**
18–49 years	Low risk	1.6	0.2	82.5	509.4	0.07	20.98	5.26	0.11	0
	At risk	10.4	2.5	661.3	1,200.5	0.22	1.81	0.00	0.65	0
	High risk	10.4	2.5	2,560.6	1,200.5	0.49	2.34	0.00	1.90	0
50–64 years	Low risk	3.2	0.6	142.9	1,046.1	0.40	20.98	5.26	0.48	0
	At risk	24.4	1.9	1,405.2	1,703.3	0.84	1.81	0.00	0.93	0
	High risk	24.4	1.9	7,898.7	1,703.3	2.38	2.34	0.00	1.98	0
65–74 years	Low risk	6.5	0.0	294.2	1,326.9	1.32	16.81	15.38	1.36	0
	At risk	46.6	2.6	2,814.3	3,296.6	1.96	25.00	32.00	2.44	0
	High risk	46.6	2.6	33,604.9	3,296.6	4.65	28.27	11.76	2.07	0
75–84 years	Low risk	8.0	0.0	630.4	1,788.2	4.10	16.81	15.38	3.16	0
	At risk	63.0	2.9	5,011.3	4,646.8	4.95	25.00	32.00	3.88	0
	High risk	63.0	2.9	31,958.8	4,646.8	7.85	28.27	11.76	4.05	0
85–99 years	Low risk	16.7	0.0	1,322.0	1,773.1	18.73	16.81	15.38	8.94	0
	At risk	98.9	0.0	10,795.0	6,895.2	14.93	25.00	32.00	11.14	0
	High risk	98.9	0.0	39,285.7	6,895.2	15.07	28.27	11.76	19.80	0

Note: The incidence of bacteraemia, meningitis and pneumonia is specified per 100,000 persons. The mortality in the general population is specified per 100 persons, whereas the mortality rates of bacteriaemia, meningitis and pneumonia are specified per 100 cases of disease. IPD: Invasive pneumococcal diseases. Sources: [21, 23, 25–28]

The mortality included both the mortality rate of the general Norwegian population and the case fatality of IPD and pneumonia. As there are no Norwegian data on the case fatality of IPD and pneumonia, these inputs were based on Danish data from the Danish National Patient Registry and included as the average case fatality in the years 2017 and 2018 [26]. Thus, it was assumed that the case fatality is similar across the two countries. The mortality is specified per 100 people for the general population and per 100 cases of disease for IPD and pneumonia. In addition, it was assumed that anyone who died would be hospitalised beforehand, thus, no mortality was assumed for pneumonia without hospitalisation. Mortality inputs of the general population and the case fatality are presented in Table 2.

### Vaccine coverage, efficacy and waning

Consistent with the study by Nymark et al., the vaccine coverage of both PCV20 and PPV23 was assumed to be 75% of the population [5]. Therefore, in the first cycle of the model, 75% of the 18–64-year-olds at risk of pneumococcal diseases and 75% of the population aged 65 years and older at either low risk or at risk of pneumococcal diseases were modelled to receive vaccination.

As has previously been confirmed by Essink et al., the immune response induced by PCV20 is non-inferior to PCV13 for all 13 serotypes [12], and so the vaccine efficacy and waning of PCV20 used in the model was assumed to be equivalent to PCV13. Therefore, PCV20 vaccine efficacy and waning was based on data from the CAPiTA study investigating people aged 65 years and older [17]. For persons aged 65 years or older at low risk or at risk, the initial PCV20 vaccine efficacy was assumed to be 45% for pneumonia and 75% for IPD. Using data from Mangen et al. on the age-specific relative changes in vaccine efficacy, the vaccine efficacy was extrapolated to people aged 50–64 years [29]. Thus, it was assumed that the initial vaccine efficacy of people aged 18–49 years was the same as persons aged 50–64 years. Based on data and post-hoc analyses of the CAPiTA study, it was assumed that the vaccine efficacy of PCV20 did not decrease within five years of vaccination [17, 30]. After five years, the annual waning of PCV20 was included in the model based on estimates by Mangen et al., who specified an annual decline in the vaccine efficacy of 5% in year 6–10, 10% in years 11–16, and after year 16, no vaccine efficacy was assumed [29].

The vaccine efficacy of PPV23 against IPD used in the model was based on data from Public Health England identified through a study by Djennad et al. [19]. To estimate the vaccine efficacy for all age groups included in the model, a logarithmic curve was fitted to the data available from Djennad et al. in the age groups 65–74 years, 75–84 years, and 85–99 years. Waning of PPV23 against IPD was also estimated based on Djennad et al. [19] with a linear decline to 76.2% of initial vaccine efficacy by year 5 followed by a linear decline to no efficacy by year 10. Thus, it was assumed that after 10 years, PPV23 had no vaccine efficacy against IPD, which is supported by Berild et al. [31]. As multiple studies have documented a lack of vaccine efficacy against pneumonia, it was assumed that PPV23 had no effect against non-bacteremic pneumonia [32–36]. An overview of the vaccine efficacy of both PPV23 and PCV20 is presented in Table 3.

**Table 3:** Vaccine efficacy against IPD and pneumonia

	IPD					Pneumococcal pneumonia				
Age/risk group	**Year 1**	**Year 5**	**Year 10**	**Year 15**	**Year 16+**	**Year 1**	**Year 5**	**Year 10**	**Year 15**	**Year 16+**
PCV20										
18–49 years										
Low risk	82%	82%	63%	37%	0%	56%	56%	43%	23%	0%
At risk	78%	78%	61%	36%	0%	53%	53%	41%	24%	0%
High risk	65%	65%	50%	30%	0%	44%	44%	34%	20%	0%
50–64 years										
Low risk	79%	79%	61%	36%	0%	51%	51%	40%	23%	0%
At risk	76%	76%	59%	35%	0%	49%	49%	38%	23%	0%
High risk	63%	63%	49%	29%	0%	41%	41%	32%	19%	0%
65 + years										
Low risk	75%	75%	58%	34%	0%	45%	45%	35%	21%	0%
At risk	72%	72%	56%	33%	0%	43%	43%	33%	20%	0%
High risk	60%	60%	46%	27%	0%	36%	36%	28%	16%	0%
PPV23										
18–49 years										
Low risk	59%	45%	0%	0%	0%	-	-	-	-	-
At risk	30%	23%	0%	0%	0%	-	-	-	-	-
High risk	17%	13%	0%	0%	0%	-	-	-	-	-
50–64 years										
Low risk	58%	44%	0%	0%	0%	-	-	-	-	-
At risk	29%	22%	0%	0%	0%	-	-	-	-	-
High risk	17%	13%	0%	0%	0%	-	-	-	-	-
65–74 years										
Low risk	56%	42%	0%	0%	0%	-	-	-	-	-
At risk	28%	21%	0%	0%	0%	-	-	-	-	-
High risk	16%	12%	0%	0%	0%	-	-	-	-	-
75–84 years										
Low risk	51%	39%	0%	0%	0%	-	-	-	-	-
At risk	25%	19%	0%	0%	0%	-	-	-	-	-
High risk	15%	11%	0%	0%	0%	-	-	-	-	-
85–99 years										
Low risk	38%	29%	0%	0%	0%	-	-	-	-	-
At risk	19%	14%	0%	0%	0%	-	-	-	-	-
High risk	11%	8%	0%	0%	0%	-	-	-	-	-

Note: The table shows the vaccine efficacy and waning of PPV23 and PCV20 against IPD and pneumonia between year 1 and 16 after vaccination. In the base case analysis, PPV23 was assumed not to have any efficacy against pneumonia. Confidence intervals are provided in parentheses. IPD: invasive pneumococcal diseases; PCV20: 20-valent pneumococcal conjugate vaccine; PPV23: 23-valent pneumococcal polysaccharide vaccine. Sources: [17, 19, 29]

For the vaccine efficacy of both PCV20 and PPV23, an adjustment was performed based on the definition of risk groups in the model, where the high-risk group only constitutes patients who currently hold a blue prescription (stem cell-transplanted, HIV-positive or with missing spleen function), meaning that the at-risk group includes immunocompromised patients who would usually be identified as high risk. Therefore, a weight was calculated representing the proportion of people in the at-risk group who would typically be categorised as high risk (20%) and multiplying it by the vaccine efficacy of high risk. The remaining proportion at risk (80%) was multiplied by the vaccine efficacy for at-risk adults.

The vaccine serotype coverage, presented in Table 4, refers to the percentage of IPD and pneumonia cases that the vaccines protect against. Based on 2019 data from the European Center for Disease prevention and Control (ECDC), it was possible to identify Norwegian data on the serotype coverage against IPD in the age groups < 1 year, 1–4 years, and ≥ 65 years [37]. Thus, no data for the age group 5–64 years was presented; however, as the total number of IPD cases was provided, it was possible to calculate the serotype coverage for this group. As it was not possible to stratify the data further into the age and risk groups used in the model, the ECDC data for 5–64-year-olds were used for the age groups of both 18–49 years and 50–64 years. In addition, the ECDC data for ≥ 65-year-olds were used for the models age groups of 65–74 years, 75–84–years, and 85–99 years. The vaccine serotype coverage for pneumonia cases was assumed to be the same as that of IPD. The IPD serotype distribution is only applied to pneumonia cases thought to be caused by *S. pneumoniae*, which is assumed to be 30% of all-cause pneumonia [5] Therefore, the IPD vaccine serotype coverage was multiplied by 30% to estimate the serotype coverage against pneumonia.

**Table 4:** Vaccine serotype coverage of PCV20 and PPV23 against IPD

Age	PCV20	PPV23
18–49 years	75.4%	79.5%
50–64 years	75.4%	79.5%
65–74 years	53.1%	66.5%
75–84 years	53.1%	66.5%
85–99 years	53.1%	66.5%

Note: The vaccine serotype coverage of PCV20 and PPV23 against IPD was based on data from 2019. IPD: invasive pneumococcal diseases; PCV20: 20-valent pneumococcal conjugate vaccine; PPV23: 23-valent pneumococcal polysaccharide vaccine. In the base case analysis, the vaccine serotype coverage against pneumonia was assumed to be identical with the one of IPD presented in this table. Source: [35]

### Herd immunity

The model included the effect of herd immunity from the childhood vaccination programme on IPD and pneumonia incidence. The effect of herd immunity was estimated based on a report by the Norwegian Institute of Public Health in which the IPD incidence before and after introduction of PCVs in the childhood vaccination programme in 2006 were provided in the population aged 65 years and older [38]. Data before introduction was based on year 2004 and 2005, when the IPD incidence was 75.6 per 100,000 persons, and which was reduced to an IPD incidence of 37.8 per 100,000 persons by 2017. Based on these estimates, it was possible to determine the annual reduction in IPD cases attributable to herd immunity during the 12.5 years (from mid-2004 to 2017), which was found to be 3.02%. As the report by the Norwegian Institute of Public Health was only based on the population aged 65 years and older, it was assumed that the estimated effect of herd immunity also applied for the population aged 18 to 64 years. In addition, the report only included the effect of herd immunity on IPD and not pneumonia; thus, it was assumed that the IPD herd immunity also applied for pneumonia. However, as *S. pneumoniae* according to Nymark et al. only accounts for 30% of all pneumonias [5], the effect of herd immunity against pneumonia only accounts for 30% of the yearly reduction in IPD cases of 3.02%, equal to a yearly reduction of pneumonia cases of 0.91%.

### Health-related quality of life

There is no health-related quality of life (HRQoL) data for the general Norwegian population which is stratified by both age and risk group. Therefore, this model used HRQoL data from a study by Ara and Brazier which investigated utility values in an English population based on age and medical history [39]. The age-specific utility data for people at low risk in the model was based on those who had no medical history, whereas a medical history of diabetes, heart attack, heart disease or hypertension was used for the at-risk population in this model. The utility data of the high-risk population was based on individuals with cancer. In Table 5, the utility values are presented based on age and risk group.

**Table 5:** Utility values based on age and risk group and disutility associated with events of IPD and pneumonia

General population utilities				Disutility due to disease			
Age	**Low risk**	**At risk**	**High risk**	**Bacteraemia**	**Meningitis**	**Pneumonia with hospital contact**	**Pneumonia without hospital contact**
18–49 years	0.9564	0.7745	0.7346	-0.13	-0.13	-0.13	-0.004
50–64 years	0.9335	0.7234	0.6852	-0.13	-0.13	-0.13	-0.004
65–74 years	0.9283	0.7308	0.7082	-0.13	-0.13	-0.13	-0.004
75–84 years	0.8921	0.6799	0.6607	-0.13	-0.13	-0.13	-0.004
85–99 years	0.8191	0.6038	0.5643	-0.13	-0.13	-0.13	-0.004

Sources: [39–41]

In the model, people who experience an event of IPD or pneumonia with hospital contact will have a reduction of the annual HRQoL of 0.13 regardless of age and risk group. This reduction is based on a study by Mangen et al., who investigated the quality of life in patients aged 65 years and older hospitalised with pneumonia in the Netherlands [40]. An event of pneumonia without hospital contact was associated with an annual reduction in HRQoL of 0.004 based on a study by Melegaro and Edmunds [41].

### Costs

As a health sector perspective was applied in the model, the analysis included costs associated with vaccination and treatment of IPD and pneumonia. All costs are presented in euros (EUR) using the exchange rate of 0.0966 from Norwegian kroner (NOK) to EUR based on the average exchange rate between 6 and 2022 and 6 December 2022 [42]. All costs are presented at the 2022 price level. Costs included in the model are presented in Table 6.

**Table 6:** Vaccination and treatment costs

	DRG-code	Unit prices, EUR	Sources
Vaccination costs			
PCV20	-	77.08	[14]
PPV23	-	26.62	[14]
Administration costs	-	46.96	[5]
Treatment costs			
Bacteraemia	416 N	11,454.45	[43]
Meningitis	20	12,386.31	[43]
Pneumonia with hospital contact (At risk and High risk)	89	5,909.44	[43]
Pneumonia with hospital contact (Low risk)	90	4,133.38	[43]
Pneumonia without hospital contact	-	45.32	[5, 44]

Note: Overview of the costs included in the model. DRG: diagnosis-related groups; PCV20: 20-valent pneumococcal conjugate vaccine; PPV23: 23-valent pneumococcal polysaccharide vaccine

The vaccination cost included both the price of the vaccine and administration costs. The vaccine prices were identified through Legemiddelsok.no in October 2022 [14], based on the maximum pharmacy retail price excluding the value added tax (VAT), to comply with guidelines from the Norwegian Medicines Agency [24]. Administration costs included the fee-for-service of a general practitioner (GP) consultations, which is calculated by multiplying the remuneration amount from “Normaltariffen” by two to comply with guidelines from the Norwegian Medicines Agency [24] and the cost of one subcutaneous injection. Based on the closed cohort, all vaccination costs occurred in the beginning of the model.

All treatment of IPD and pneumonia with hospital contact were assumed to occur within the hospital sector, assuming no outpatient care. All costs associated with inpatient care were identified using the Norwegian diagnosis-related group (DRG) tariffs [43]. Patients experiencing events of pneumonia without hospital contact received outpatient care, in which one GP visit and the costs of antibiotics were included based on the studies by Nymark et al. and Wollf et al. [5, 44].

### Sensitivity analyses

The uncertainty of the model was investigated in one-way sensitivity analyses (OWSA), scenario analyses and probabilistic sensitivity analysis (PSA). In the OWSA, the uncertainty of input parameters and methodological assumptions were evaluated by varying each parameter one at time, using a ± 20% for inputs for disease incidence, mortality, vaccine efficacy of PCV20 and PPV23, and costs. For the remaining inputs of utility, disutility, proportion of pneumonia due to *S. pneumonia* and the proportion of IPD due to vaccine serotypes, the uncertainty was investigated with ± 10%.

Multiple scenario analyses were conducted to evaluate the impact of alternative inputs of the methodological assumptions regarding the vaccine efficacy of PPV23 against pneumonia, vaccine coverage, usage of the IPD distribution of serotype coverage for pneumonia, the length of the time horizon (5 and 10 years), the discount rate (0% and 7%), the disutility of IPD and the choice of comparator on the base case results. The scenario analysis including the vaccine efficacy of PPV23 against pneumonia used inputs from a study by Lawrence et al., who found an efficacy of 25.7% among persons aged 16–74 years and 4.7% among persons aged 75 years and older [45]. The vaccine coverage was assumed to be 75% based on Nymark et al. However, this assumption was associated with uncertainty, as the data from the Norwegian Immunisation Registry SYSVAK and NPR indicated a lower pneumococcal vaccine coverage [23, 25]. Therefore, scenario analyses with the vaccine coverage set to 25%, 50% and 100% were conducted. Scenario analyses were also performed to investigate the impact of serotype distribution and coverage of PCV20 on the number of pneumonia cases requiring hospitalisation. Data regarding the percentage of pneumonia caused by serotypes of *S. pneumoniae*, which are covered by PCV20 used for the sensitivity analyses were based on a Danish study by Benfield et al. and a Swedish study by Theilacker et al. [46, 47]. The serotype distributions of Benfield et al. and Theilacker et al. were adjusted by the 30% of pneumonia cases that are caused by *S. pneumoniae.* Therefore, the PCV20 serotype coverage was changed to 15.2% for patients aged 65 years and older and 20.7% for patients aged 18–64 years using Theilacker et al. and 16.9% using Benfield et al. [46, 47]. A scenario analysis in which the disutility of IPD was increased by 100% to capture that IPD is assumed to constitute more severe illness than pneumonia with hospital contact was also conducted. In addition, a scenario analysis comparing PCV20 with no vaccination was included to investigate PCV20 to the current standard practice of vaccination against pneumococcal diseases in Norway. Finally, a scenario assuming linear waning to 0% between years 5 and 15 for PCV20 and a scenario setting the herd immunity effect to 0% were included.

To assess the joint uncertainty of the input parameters, a probabilistic sensitivity analysis (PSA) with 1,000 iterations was performed. A normal distribution was used for input parameters related to disease incidence, mortality and the vaccine prices. Beta distributions were used for parameters of utility, disutility, vaccine efficacy, the proportion of pneumonia due to *S. pneumoniae* and the proportion of IPD vaccine serotypes. Costs related to treatment were included in the PSA using a gamma distribution. Standard deviations for the applied distributions were derived.

## Results

Results of the base case analysis are shown in Table 7. The results indicate that PCV20 is associated with fewer cases of pneumococcal diseases and fewer associated deaths compared to PPV23 using a lifetime time horizon of 81 years. PCV20 resulted in 1,539 fewer cases of bacteriaemia, 98 fewer cases of meningitis, 26,867 fewer cases of pneumonia with hospital contact, and 30,149 fewer cases of pneumonia without hospital contact compared to vaccination with PPV23. Similarly, PCV20 resulted in 330 and 1,055 fewer deaths due to IPD and pneumonia, respectively. In addition, the life year gain was 7,584 higher and the QALY gain was 7,966 higher for PCV20 than PPV23.

**Table 7:** Base case results

	PPV23	PCV20	Increments
Clinical outcomes			
Number of IPD cases			
⋅ Bacteraemia	28,602	27,064	-1,539
⋅ Meningitis	1,345	1,247	-98
Number of pneumonia cases			
⋅ With hospital contact	2,255,906	2,229,039	-26,867
⋅ Without hospital contact	2,794,766	2,764,617	-30,149
Number of deaths due to IPD	7,004	6,674	-330
Number of deaths due to pneumonia	122,412	121,357	-1,055
Life years gained	70,392,931	70,400,516	7,584
⋅ Life years per person	16.77	16.77	0.0018
QALYs gained	60,123,775	60,131,741	7,966
⋅ QALYs per person	14.32	14.33	0.0019
Economic outcomes (EUR)			
Total costs	5,599,670,010	5,526,062,665	-73,607,345
⋅ Total costs per person	1,334	1,317	-17.54
Healthcare costs			
⋅ Vaccination costs	35,453,377	102,654,202	67,200,826
⋅ Hospital sector	5,445,778,380	5,306,065,869	-139,712,512
⋅ Primary sector	118,438,253	117,342,594	-1,095,659
Cost-effectiveness			
⋅ Incremental costs per life year gained	-9,705 (PCV20 is the dominant strategy)		
⋅ Incremental costs per QALY gained	-9,240 (PSV20 is the dominant strategy)		

Note: The table shows the clinical and economic outcomes of the base case analysis for each vaccination strategy and the increments between the strategies. Both the accumulated and per person QALYs, life years and total costs are presented. QALY: Quality-adjusted life years; ICER: Incremental cost-effectiveness ratio; IPD: Invasive pneumococcal diseases; PCV20: 20-valent pneumococcal conjugate vaccine; PPV23: 23-valent pneumococcal polysaccharide vaccine

PCV20 is a more costly vaccine than PPV23; therefore, the cost of vaccination was EUR 67,200,826 higher when vaccinating with PCV20. When costs associated with treatment of IPD and pneumonia at the hospital and primary sector were investigated, it was found that PCV20 resulted in a lower cost of EUR 139,712,512 in hospital costs and EUR 1,095,659 in primary sector costs than PPV23. This resulted in PCV20 having a total cost that was EUR 73,607,345 lower than PPV23.

As PCV20 resulted in a higher QALY gain at a lower total cost compared to PPV23, the estimated incremental cost-effectiveness ratio (ICER) was negative (-9,240 EUR per QALY gained), indicating that PCV20 is the dominant vaccine strategy compared to PPV23.

### Sensitivity analyses

**Fig. 2:**
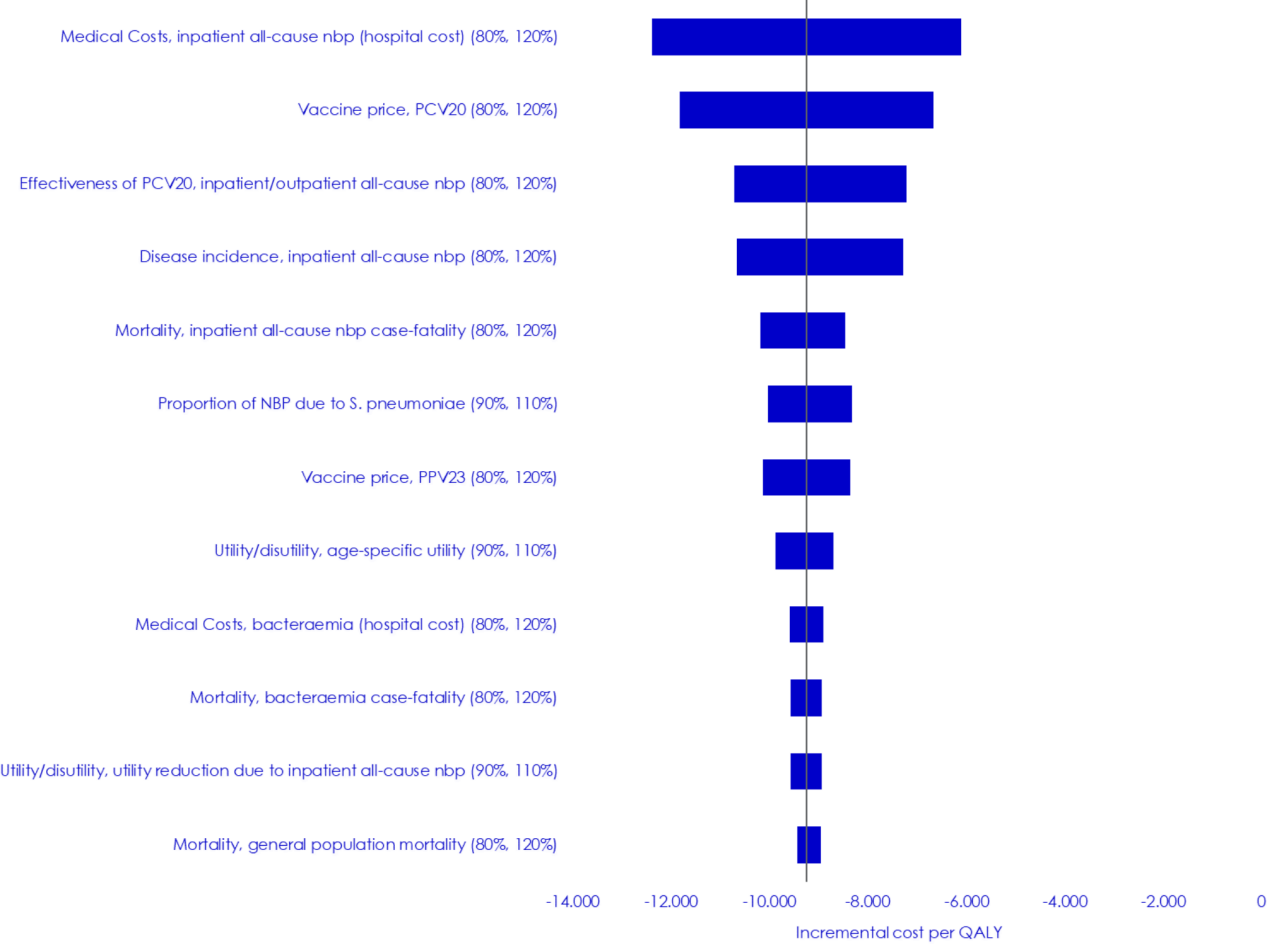
Tornado diagram showing the 12 input parameters with the highest impact on the incremental cost-effectiveness ratio *Note*: NBP: Non-bacteraemic pneumonia

The OWSA showed that the results were robust to changes in the input parameters when they were changed one at a time. And thus, in all OWSA, PCV20 remained the dominant vaccine strategy when compared to PPV23. A tornado diagram of the 12 parameters with the highest impact on the result is shown in Fig. 2.

The scenario analyses showed that despite using alternative inputs for the vaccine coverage of the population, including vaccine efficacy of PPV23 against pneumonia, changing the time horizon, using different discount rates and increased disutility associated with IPD, and using different inputs for PCV20’s waning and herd immunity, the results were robust. However, when the time horizon was changed to five years or PCV20 was compared to no vaccine, the results showed that PCV20 was associated with a greater health gain than PPV23 or no vaccine but at an additional cost, resulting in positive ICER estimates of EUR 1,437 per QALY gained and EUR 2,292 per QALY gained, respectively, see Table 8.

**Table 8:** Results of scenario analyses

	Total incremental cost (EUR)	Total incremental QALYs gained	ICER (cost per QALY gained)	∆ICER (%) compared to base case
Base case analysis	**-73,607,345**	**7,966**	**-9,240**	**-**
Efficacy and waning of PPV23 vaccine using estimates by Lawrence et al.	-59,801,031	7,341	-8,146	+ 12%
Vaccine coverage 25%	-24,535,782	2,655	-9,240	-
Vaccine coverage 50%	-49,071,563	5,311	-9,240	-
Vaccine coverage 100%	-98,143,127	10,622	-9,240	-
PCV20 serotype coverage against pneumonia: Benfield et al. (16.9% for all age groups)	-69,346,273	7,881	-8,799	+ 5%
PCV20 serotype coverage against pneumonia: Theilacker et al. (20.7% 18–64 years and 15.2% +65 years)	-66,352,656	7,615	-8,713	+ 6%
Time horizon 5 years	3,027,134	2,107	1,437	+ 116%
Time horizon 10 years	-45,175,157	4,474	-10,098	-9%
Discount rate 0%	-109,818,187	12,119	-9,062	+ 2%
Discount rate 7%	-53,778,375	6,185	-8,695	+ 6%
Disutility of IPD increased by 100%	-73,607,345	8,097	-9,091	+ 2%
PCV20 compared with no vaccination	19,529,114	8,519	2,292	+ 125%
PCV 20 linear waning to 0%, year 5–15	-48.964.932	6.468	-7,570	+ 18%
Herd immunity effect set to 0%	-75,156,959	8,017	-9,313	-1%

Note: The table shows the results of the scenario analyses according to the total incremental costs and QALYs between PCV20 and PPV23 and the calculated ICER

ICER: Incremental cost-effectiveness ratio; QALY: Quality-adjusted life years; IPD: Invasive pneumococcal diseases; PCV20: 20-valent pneumococcal conjugate vaccine; PPV23: 23-valent pneumococcal polysaccharide vaccine

**Fig. 3:**
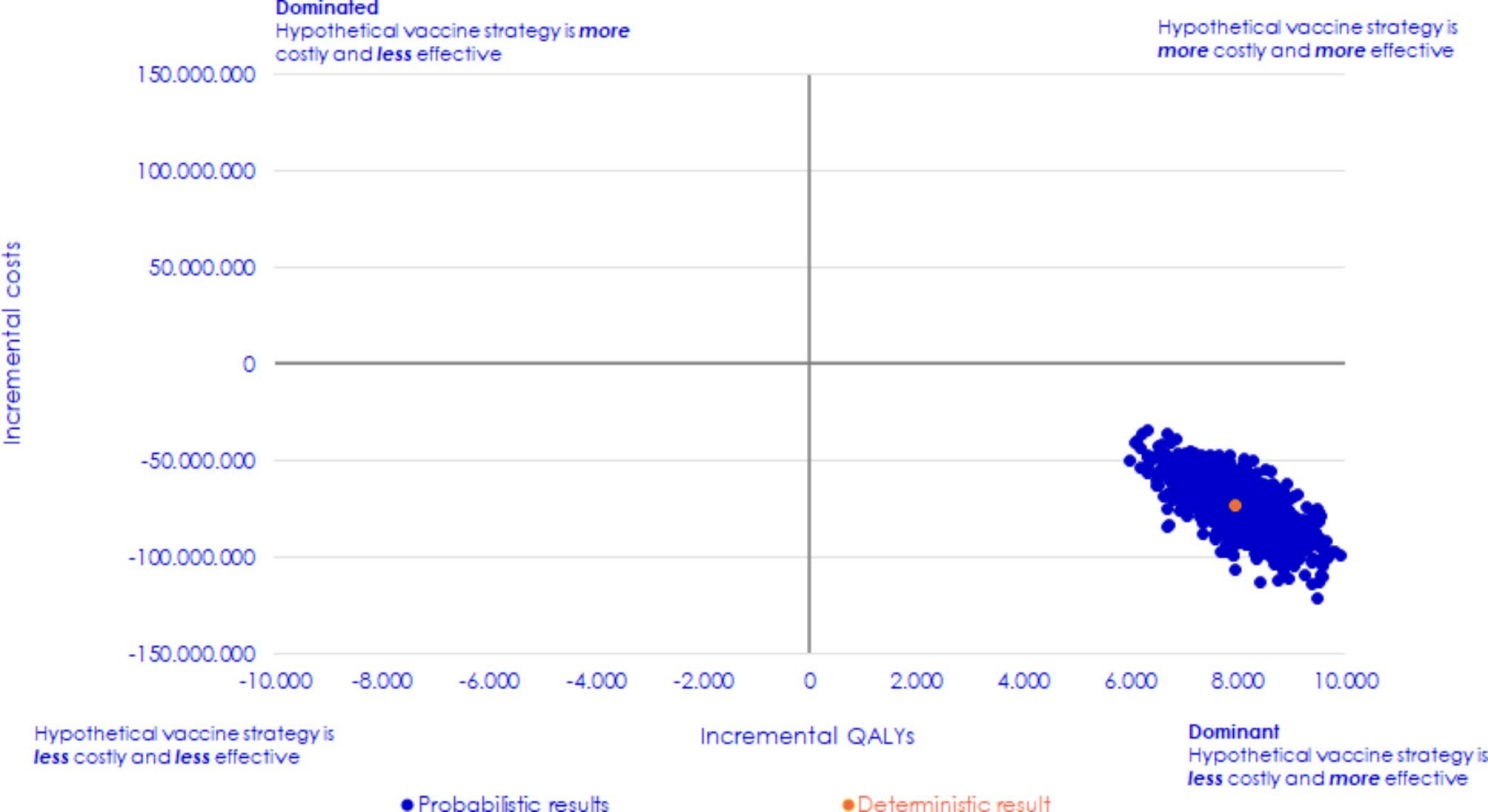
Results of the probabilistic sensitivity analysis illustrated in a cost-effectiveness plane

Results of the PSA are illustrated in an ICER plane in Fig. 3. The ICER plane shows that in the 1,000 iterations performed during the PSA, 100% resulted in PCV20 having a greater QALY gain at a lower cost compared to PPV23; thus, PCV20 remained the dominant strategy in all iterations. Based on the 1,000 iterations of the PSA, the average incremental QALYs were estimated to 7,990 and the average incremental costs were estimated to EUR − 73,198,717, resulting in an ICER of EUR − 9,161 per QALY gained.

## Discussion

This study investigated the health benefits and costs of vaccinating Norwegian adults aged 18 years and older at risk of pneumococcal diseases and adults aged 65 years and older at low risk or at risk of pneumococcal diseases with PCV20 compared to PPV23. The results emphasised that PCV20 was associated with a higher QALY gain and lower total costs than PPV23, and thus, PCV20 constituted the dominant vaccine strategy. The robustness of the results was evaluated through multiple sensitivity analyses, of which the majority showed that PCV20 remained the dominant vaccine strategy. Similar results of PCV20 being the dominant vaccine strategy compared to PPV23 have been identified in an English setting by Mendes et al., who evaluated the cost-effectiveness in adults aged 18 to 64 years with underlying conditions and all adults aged 65 to 99 years [48]. Thus, even though the serotype coverage is better for PPV23 (four more serotypes than PCV20) the outcomes are better for PCV20 as PCV20’s efficacy is expected to be higher, its duration of protection longer, and to confer protection against non-bacteremic pneumonia similarly to PCV13. These attributes outweigh the serotype gap among adults in Norway. Indeed, while there is PPV23-type disease not preventable by PCV20, the majority is non-bacteremic pneumonia which is also not preventable by PPV23.

However, when the time horizon was reduced to five years or when PCV20 was compared to no vaccine, it resulted in positive ICERs, indicating that PCV20 is associated with a greater gain in health at a higher cost than the comparator. As there is no official cost-effectiveness threshold in Norway, it is not possible to determine which of the vaccine strategies would be deemed cost-effective. Despite this, it should be emphasised that the ICERs were very low (EUR 1,437 and EUR 2,292 per QALY gained) in both the scenario of a short time horizon and when compared to no vaccine.

The cost-effectiveness of implementing a universal pneumococcal vaccination programme of PPV23 for older adults in Norway has previously been investigated by Nymark et al., who found that a universal vaccination programme was expected to be cost-effective among those older than 75 years (ICER in the lower end of the cost-effectiveness threshold range). Among those older than 65 years a universal vaccination programme was likely to be cost-effective according to Nymark et al. [5]. Therefore, it should be emphasised that implementing PCV20 in a universal pneumococcal vaccination programme in Norway could be favourable, as the current study found PCV20 to be cost-effective compared to PPV23. The cost-effectiveness of implementing PCV20 in a universal vaccination programme was also investigated in a scenario analysis that compared PCV20 to no vaccine, which is the current standard practice in Norway. This scenario analysis showed that PCV20 was associated with an additional cost of EUR 2,292 per extra QALY gained compared to no vaccine. As it is a low ICER, it is possible that PCV20 would be deemed cost-effective. Notably, an ICER of NOK 275,000 (EUR 26,572) per QALY gained was assumed cost-effective even at the lowest level of disease severity by a task force formed by the Norwegian Ministry of Health and Welfare in 2015 [49], well above the ICER identified in this study. Further in 2010, the Norwegian Medicines Agency assessed that HPV vaccination of 14–16 years old girls were cost-effective with an estimated ICER of NOK 48,000 (4,638 EUR) per QALY [50].

Differences in the efficacy and effectiveness of PPV23 and PCV20 have been observed, especially with regard to the protection against pneumonia, in which the effectiveness of PCV vaccines have been identified as high as 72.8%, whereas PPV23 has showed either little or no effect by offering only 2–3% protection [13, 51–53]. For the base case analysis, no vaccine efficacy of PPV23 against pneumonia was assumed. Therefore, the higher vaccine efficacy of PCV20 against pneumonia used in this model results in better avoidance of pneumonia cases with PCV20. Using different studies of the vaccine efficacy of PPV23 against pneumonia could result in different results on cost-effectiveness. However, when the vaccine efficacy of PPV23 against pneumonia was changed to that found by Lawrence et al. [45], the cost-effectiveness was re-evaluated and showed that PCV20 remained dominant.

The assumption that the serotype distribution of pneumonia is identical with that of IPD was investigated in a series of sensitivity analyses. The purpose of this was to investigate the impact of the serotype distribution and serotype coverage of PCV20 on the number of pneumonia cases requiring hospitalisation compared to the base case. When the serotype coverage was changed to both a higher and lower level than the base case, PCV20 remained dominant, and thus, the assumption did not substantially impact the cost-effectiveness results.

The percentage of cases due to vaccine serotypes was assumed to be constant over time which is a limitation as paediatric introduction of PCV15 or higher valency PCVs not yet available may lead to serotype replacement. Predicting future serotype replacement is challenging and was not attempted in the current analysis. However, only replacement by the four PPV23 serotypes not contained in PCV20 would be expected to affect the ICER in the comparison against PPV23.

The assumption of a 75% vaccine coverage was based on Nymark et al., who assumed a 75% vaccination coverage among adults aged 65 years [5]. Therefore, it is possible that the vaccine coverage among adults aged 18–64 years is overestimated. In general, the vaccine coverage used in this analysis was high, as the pneumococcal vaccine uptake among adults in Norway are approximately 15% [15], indicating that the reality in Norway differs considerably from the base case analysis of this model, in which a 75% vaccine coverage was assumed. The use of a higher vaccine coverage is also supported by the current vaccine coverage of COVID-19 and influenza in Norway, which is above 90% and 62%, respectively [54]. As the vaccine coverage is the same for PCV20 and PPV23, using a higher coverage will impact the results by resulting in both higher health gains and costs. When the impact of vaccine coverage was investigated through multiple scenario sensitivity analyses, the ICER remained negative, with PCV20 being the dominant vaccine strategy.

Inputs for the model regarding the disease incidence and mortality rate of the general Norwegian population at low risk were based on real-world evidence from Statistics Norway and Norwegian patient registries. When registry data is used it is important to emphasise that it only reflects the data, which has been reported. Therefore, it is possible that the registry data is not the exact truth. However, the use of registry data is a strength, as it ensures that the model illustrates a Norwegian setting. As Norwegian data was not available for all inputs, it was necessary to use foreign data, which can be a limitation, as transferability of data across countries can be questionable due to differences in the delivery of healthcare and demographics of the population [55]. The data regarding mortality was based on Danish real-world evidence, which was used due to generally similar populations in the Nordic countries. When Danish mortality data on people at low risk were compared to that of Norway, the data were found to correspond well. However, as risk groups are defined differently in Norway and Denmark, cf. the Danish adaption of the model [20], the Danish mortality rates applied in the Norwegian model adaption could result in either higher or lower mortality estimates.

The utility data used in the model were based on the study by Ara and Brazier and an English population [39]. This study was used to include utility values stratified by both age and risk groups, as there are no available Norwegian utility data which are stratified by risk groups. The use of utility values from Ara and Brazier was validated by comparing the values found by Ara and Brazier in the no-risk group with the Norwegian age-specific utility values used by the Norwegian Medicines Agency [24]. A great similarity between the utility values of the age groups was found; however, a general tendency of the Norwegian utility values being slightly lower than the English values from Ara and Brazier was identified, i.e., the population aged 71–80 years in Norway has a utility value of 0.808. This difference could be explained by the Norwegian utility values representing the general population across all risk groups. Therefore, the Norwegian utility values represent a weighted average across all risk groups.

The utility values of the high-risk group was, as stated, based on people with cancer in Ara and Brazier [39]. However, this does not match the description of high-risk patients in the Norwegian adaption, as the high-risk group only comprises patients who currently hold a blue prescription. The decision to base high-risk utility values on cancer is based on the available literature from Ara and Brazier, in which cancer represents the best knowledge of a disease that is usually categorised as high risk, even though it is not included as high risk in the current model. This is not expected to impact the results, as the main difference in utility values of the risk groups is seen between low risk and at risk, not at risk and high risk, indicating that it will not affect the model outcomes to use cancer as a proxy for high risk. In addition, the at-risk utility values were based on only a few diseases, i.e., diabetes, heart attack, hypertension, and other heart diseases. Therefore, the at-risk utility values are estimated on a limited number of diseases categorised as at risk and is potentially overestimated or underestimated.

The model included conservative assumptions for both costs and utility inputs, all of which could influence the results by either overestimating or underestimating the cost-effectiveness of PCV20 and were therefore assessed in sensitivity analyses. One of the conservative assumptions was that the disutility of IPD was the same as for pneumonia with hospital contact. In general, it would be expected that IPD is associated with a higher level of disutility than pneumonia with hospital contact, as IPD is expected to constitute more severe diseases than pneumonia. This assumption was investigated in a sensitivity analysis in which the disutility of IPD was increased by 100%, and the analysis showed that PCV20 remained the dominant vaccine strategy. Therefore, the decision to use a conservative input of IPD disutility did not influence the results of the analysis. Another conservative assumption included was to exclude revaccination of PPV23. According to the guidelines of the Norwegian Institute of Public Health, people should be revaccinated with PPV23 every six years, whereas no revaccination of PCV20 is needed [11]. Therefore, the exclusion of PPV23 revaccination resulted in a lower vaccination cost of PPV23. However, the inclusion would only have increased the cost-effectiveness of PCV20.

The model used a health-service perspective; therefore, the analysis did not include costs of patient time and transportation or any indirect costs from productivity loss. However, according to guidelines from the Norwegian Medicines Agency, an extended health-service perspective should be used, and therefore, both patient time and transportation should be included [24]. These costs were excluded based on lack of data of number of contacts that patients have along with the travel time.

## Conclusion

Results of this study showed that vaccinating the Norwegian population aged 18 to 99 years at risk of pneumococcal diseases and the population aged 65 years and older at low risk with PCV20 was cost-effective compared to PPV23. The anticipated reduction in cases of pneumococcal diseases and deaths would offset the incremental cost of PCV20 through reduction in treatment costs, and this result of PCV20 being the dominant vaccine strategy remained consistent through numerous sensitivity analyses; the few scenarios which indicated PCV20 would incur little incremental cost for the health benefits accrued. Broadening access to adult pneumococcal conjugate vaccines beyond the highest-risk patients in Norway should be considered.

## Data Availability

Input data used in the model are publicly available or can be retrieved from Norwegian National Patient Registry, Norwegian Registry for Primary Care, Statistics Norway, and other publicly available sources. The model will not be shared.

## Declarations

### Disclaimer

Information from the Norwegian Patient Registry (NPR), Norwegian Registry for Primary Care (KPR) and the Norwegian surveillance system for communicable diseases registry (MSIS) has been used in this publication. The interpretation and reporting of these data are the sole responsibility of the authors, and no endorsement by the NPR, KPR and MSIS is intended nor should be inferred.

## Abbreviations

CAP: Community-acquired pneumonia
CAPiTA: Community-Acquired Pneumonia Immunization Trial in Adults
DRG: Diagnosis-related groups
DSA: Deterministic sensitivity analysis
ECDC: European Centre for Disease prevention and Control
EUR: Euros
GP: General practitioner
HRQoL: Health-related quality of life
ICD-10: 10th edition of the International Classification of Diseases
IPD: Invasive pneumococcal diseases
MSIS: Norwegian surveillance system for communicable diseases registry
NBP: Non-bacteraemic pneumonia
NOK: Norwegian kroner
NPR: Norwegian National Patient Registry
OWSA: One-way sensitivity analyses
PCV: Pneumococcal conjugate vaccine
PCV7: 7-valent pneumococcal conjugate vaccine
PCV13: 13-valent pneumococcal conjugate vaccine
PCV15: 15-valent pneumococcal conjugate vaccine
PCV20: 20-valent pneumococcal conjugate vaccine
PPV23: 23-valent pneumococcal polysaccharide vaccine
PSA: Probabilistic sensitivity analysis
VAT: Value added tax

## References

[1] Drijkoningen JJC, Rohde GGU (2014). Pneumococcal infection in adults: burden of disease. Clin Microbiol Infect.

[2] Theilacker C, Fletcher M, Jodar L (2022). PCV13 vaccination of adults against Pneumococcal Disease: what we have learned from the community-acquired pneumonia immunization trial in adults (CAPiTA). Microorganisms.

[3] Plotkin SA, Orenstein WA, Offit PA (2018). Plotkin’s vaccines, chap. 46–47. Seventh edition.

[4] Winje BA, Berild JD, Vestrheim DF (2022). Efficacy and effectiveness of pneumococcal vaccination in adults – a second update of the literature [Effekt av pneumokokkvacsine hos eldre].

[5] Nymark LS, Dag Berild J, Lyngstad TM et al. Cost-utility analysis of the universal pneumococcal vaccination programme for older adults in Norway. Hum Vaccines Immunother. 2022;2101333. 10.1080/21645515.2022.2101333 PMC9746426 35917277

[6] Ganaie F, Saad JS, McGee L (2020). A New Pneumococcal Capsule Type, 10D, is the 100th serotype and has a large cps fragment from an oral Streptococcus. mBio.

[7] Blasi F, Mantero M, Santus P (2012). Understanding the burden of pneumococcal disease in adults. Clin Microbiol Infect.

[8] Welte T, Torres A, Nathwani D (2012). Clinical and economic burden of community-acquired pneumonia among adults in Europe. Thorax.

[9] Golos M, Eliakim-Raz N, Stern A et al. Conjugated pneumococcal vaccine versus polysaccharide pneumococcal vaccine for prevention of pneumonia and invasive pneumococcal disease in immunocompetent and immunocompromised adults and children. Cochrane Acute Respiratory Infections Group, editor. Cochrane Database Syst Rev [Internet]. 2019 [cited 2022 Oct 31]; Available from: 10.1002/14651858.CD012306.pub2.

[10] Pollard AJ, Perrett KP, Beverley PC (2009). Maintaining protection against invasive bacteria with protein–polysaccharide conjugate vaccines. Nat Rev Immunol.

[11] Folkehelseinstituttet (FHI) [Norwegian Institute of Public Health]. Pneumokokkvaksine - veileder for helsepersonell [Pneumococcal vaccine - guide for healthcare personnel] [Internet]. 2022. Available from: https://www.fhi.no/nettpub/vaksinasjonsveilederen-for-helsepersonell/vaksiner-mot-de-enkelte-sykdommene/pneumokokkvaksinasjon---veileder-fo/#pneumokokkvaksiner.

[12] Essink B, Sabharwal C, Cannon K et al. Pivotal phase 3 Randomized Clinical Trial of the Safety, Tolerability, and immunogenicity of 20-Valent Pneumococcal Conjugate vaccine in adults aged ≥ 18 years. Clin Infect Dis. 2021;ciab990. 10.1093/cid/ciab990 PMC9427137 34940806

[13] Dunne EM, Cilloniz C, von Mollendorf C et al. Pneumococcal vaccination in adults: what can we learn from Observational Studies that evaluated PCV13 and PPV23 effectiveness in the same Population? Arch Bronconeumol. 2023;S0300289623000030. 10.1016/j.arbres.2022.12.015 36681604

[14] Legemiddelsok.no. Legemiddelsok.no (2022) [Internet]. 2022. Available from: https://www.legemiddelsok.no/.

[15] Winje BA, Vestrheim DF, White RA (2021). The risk of Invasive Pneumococcal Disease differs between risk groups in Norway following widespread use of the 13-Valent Pneumococcal Vaccine in Children. Microorganisms.

[16] Helse- og omsorgsdepartementet. Forskrift om stønad til dekning av utgifter til viktige legemidler mv. (blåreseptforskriften) [Regulation on benefits to cover expenses for important medicines etc. (blue prescription regulation)] [Internet]. 2022. Available from: https://lovdata.no/dokument/SF/forskrift/2007-06-28-814.

[17] Bonten MJM, Huijts SM, Bolkenbaas M (2015). Polysaccharide Conjugate Vaccine against Pneumococcal Pneumonia in adults. N Engl J Med.

[18] Andrews NJ, Waight PA, George RC (2012). Impact and effectiveness of 23-valent pneumococcal polysaccharide vaccine against invasive pneumococcal disease in the elderly in England and Wales. Vaccine.

[19] Djennad A, Ramsay ME, Pebody R (2018). Effectiveness of 23-Valent Polysaccharide Pneumococcal Vaccine and Changes in Invasive Pneumococcal Disease incidence from 2000 to 2017 in those aged 65 and over in England and Wales. EClinicalMedicine.

[20] Olsen J, Schnack H, Skovdal M et al. Cost-effectiveness of 20-valent pneumococcal conjugate vaccine in Denmark compared with PPV23. J Med Econ. 2022;1–49. 10.1080/13696998.2022.2152235 36426797

[21] Statistisk sentralbyrå [Statistics Norway]. 07459: Alders- og kjønnsfordeling i kommuner, fylker og hele landets befolkning (K) 1986–2022 [07459: Population, by sex and one-year age groups (M) 1986–2022] [Internet]. 2019. Available from: https://www.ssb.no/statbank/table/07459/.

[22] Folkehelseinstituttet (FHI) [Norwegian Institute of Public Health]. Meldingssystem for smittsomme sykdommer (MSIS) [Norwegian Surveillance System for Communicable Diseases (MSIS)] [Internet]. 2022. Available from: https://www.fhi.no/hn/helseregistre-og-registre/msis/.

[23] Helsedirektoratet [Norwegian Directorate of Health]. Norsk pasientregister (NPR) [Norwegian Patient Registry] [Internet]. 2019 [cited 2022 Oct 20]. Available from: https://www.helsedirektoratet.no/tema/statistikk-registre-og-rapporter/helsedata-og-helseregistre/norsk-pasientregister-npr.

[24] Statens Legemiddelverk (NoMA). Guidelines for the submission of documentation for single technology assessment (STA) of pharmaceuticals [Internet]. 2021. Available from: https://legemiddelverket.no/Documents/English/Public%20funding%20and%20pricing/Documentation%20for%20STA/Guidelines%2018.10.2021.pdf.

[25] Helsedirektoratet [Norwegian Directorate of Health]. Kommunalt pasient- og brukerregister (KPR) [Norwegian Register of Primary Health Care] [Internet]. 2019 [cited 2022 Oct 20]. Available from: https://www.fhi.no/en/more/access-to-data/about-the-national-health-registries2/.

[26] Danmarks Statistik [Statistics Denmark]. Deaths by sex, age and cause of death (Table DOD1) [Internet]. [cited 2022 Jan 15]. Available from: https://www.statistikbanken.dk/DOD1.

[27] Sundhedsdatastyrelsen [The Danish Health Data Authority]. Data extracted from the Danish National Patient Registry. 2020.

[28] Sundhedsdatastyrelsen [The Danish Health Data Authority]. Data extracted from the Danish Cause of Death Registry. 2020.

[29] Mangen M-JJ, Rozenbaum MH, Huijts SM (2015). Cost-effectiveness of adult pneumococcal conjugate vaccination in the Netherlands. Eur Respir J.

[30] Patterson S, Webber C, Patton M (2016). A post hoc assessment of duration of protection in CAPiTA (Community Acquired Pneumonia immunization trial in adults). Trials Vaccinol.

[31] Berild JD, Winje BA, Vestrheim DF (2020). A systematic review of studies published between 2016 and 2019 on the effectiveness and efficacy of pneumococcal vaccination on Pneumonia and Invasive Pneumococcal Disease in an Elderly Population. Pathogens.

[32] Moberley S, Holden J, Tatham DP, Vaccines for preventing pneumococcal infection in adults [Internet]., Chichester et al. UK: John Wiley & Sons, Ltd; 2008 [cited 2022 Feb 23]. p. CD000422.pub2. Available from: 10.1002/14651858.CD000422.pub2. 10.1002/14651858.CD000422.pub2 18253977

[33] Tin Tin Htar M, Stuurman AL, Ferreira G et al. Effectiveness of pneumococcal vaccines in preventing pneumonia in adults, a systematic review and meta-analyses of observational studies. Arez AP, editor. PLOS ONE. 2017;12:e0177985. 10.1371/journal.pone.0177985 PMC5441633 28542347

[34] Latifi-Navid H, Latifi-Navid S, Mostafaiy B (2018). Pneumococcal Disease and the effectiveness of the PPV23 vaccine in adults: a two-stage bayesian Meta-analysis of Observational and RCT Reports. Sci Rep.

[35] Heo JY, Seo YB, Choi WS et al. Effectiveness of pneumococcal vaccination against pneumococcal pneumonia hospitalization in older adults: a prospective, test-negative study. J Infect Dis. 2021;jiab474. 10.1093/infdis/jiab474 34537847

[36] Kim JH, Chun BC, Song JY (2019). Direct effectiveness of pneumococcal polysaccharide vaccine against invasive pneumococcal disease and non-bacteremic pneumococcal pneumonia in elderly population in the era of pneumococcal conjugate vaccine: a case-control study. Vaccine.

[37] Disease data for pneumococcal disease from ECDC Surveillance Atlas [Internet]. ECDC. ; 2019. Available from: https://www.ecdc.europa.eu/en/pneumococcal-disease/surveillance-and-disease-data/atlas.

[38] Berg AS, Berild JD, Caugant DA, et al. Årsrapport 2017 - invasive infeksjoner. Folkehelseinstitutet; 2018.

[39] Ara R, Brazier JE (2011). Using Health State Utility values from the General Population to approximate baselines in decision Analytic Models when Condition-Specific data are not available. Value Health.

[40] Mangen M-JJ, Huijts SM, Bonten MJM (2017). The impact of community-acquired pneumonia on the health-related quality-of-life in elderly. BMC Infect Dis.

[41] Melegaro A, Edmunds WJ (2004). Cost-effectiveness analysis of pneumococcal conjugate vaccination in England and Wales. Vaccine.

[42] Nationalbankens Statistiskbank [the Nationalbank’s statistic bank]. Valutakurser fra DNVALD NOK til EUR (6. oktober 2022 til 6. december 2022) [Exchange rate from DNVALD NOK to EUR (6 October 2022 to 6 December 2022)] [Internet]. 2022. Available from: https://www.nationalbanken.dk/valutakurser.

[43] Helsedirektoratet [Norwegian Directorate of Health]. Norwegian DRG tariffs - Innsatsstyrt finansiering (ISF) [Internet]. 2022. Available from: https://www.helsedirektoratet.no/tema/finansiering/innsatsstyrt-finansiering-og-drg-systemet/innsatsstyrt-finansiering-isf#forelopigisfregelverk.

[44] Wolff E, Storsaeter J, Örtqvist Ã (2020). Cost-effectiveness of pneumococcal vaccination for elderly in Sweden. Vaccine.

[45] Lawrence H, Pick H, Baskaran V et al. Effectiveness of the 23-valent pneumococcal polysaccharide vaccine against vaccine serotype pneumococcal pneumonia in adults: A case-control test-negative design study. Kretzschmar MEE, editor. PLOS Med. 2020;17:e1003326. 10.1371/journal.pmed.1003326 PMC7584218 33095759

[46] Benfield T, Skovgaard M, Schønheyder HC et al. Serotype Distribution in Non-Bacteremic Pneumococcal Pneumonia: Association with Disease Severity and Implications for Pneumococcal Conjugate Vaccines. Beall B, editor. PLoS ONE. 2013;8:e72743. 10.1371/journal.pone.0072743 PMC3751823 24009703

[47] Theilacker C. Pneumococcal serotype distribution in adults hospitalized with radiologically-confirmed community-aquired pneumonia in Malmö, Sweden [Internet]. 2020. Available from: https://cslide.ctimeetingtech.com/isppd20/attendee/eposter/poster/904?q=Theilacker.

[48] Mendes D, Averin A, Atwood M (2022). Cost-effectiveness of using a 20-valent pneumococcal conjugate vaccine to directly protect adults in England at elevated risk of pneumococcal disease. Expert Rev Pharmacoecon Outcomes Res.

[49] Helse- og omsorgsdepartementet [the Norwegain Ministry of health and Welfare]. På ramme alvor - Alvorlighet og prioritering [Severity of illness and priority setting in Norway]. 2015.

[50] Nymark L, Winje B, Berild J et al. Pneumokokkvaksine til eldre som vaksinasjonsprogram [Pneumococcal vaccine to elderly in a national immunization program] [Internet]. Folkehelseinstituttet; 2023. Available from: https://www.fhi.no/contentassets/668bc45114364413ab2f032d076c95c3/metodevurdering-pneumokokkvaksine-til-eldre.pdf.

[51] Chandler T, Furmanek S, Carrico R (2022). 23-Valent Pneumococcal Polysaccharide Vaccination does not prevent community-acquired pneumonia hospitalizations due to vaccine-type Streptococcus pneumoniae. Microorganisms.

[52] Kolditz M, Schmitt J, Pletz MW (2019). Impact of the 13-Valent Pneumococcal Conjugate Vaccine on the incidence of all-cause pneumonia in adults aged ≥ 60 years: a Population-based, Retrospective Cohort Study. Clin Infect Dis.

[53] McLaughlin JM, Jiang Q, Isturiz RE et al. Effectiveness of 13-Valent Pneumococcal Conjugate Vaccine Against Hospitalization for Community-Acquired Pneumonia in Older US Adults: A Test-Negative Design. Clin Infect Dis [Internet]. 2018 [cited 2022 Oct 17]; Available from: https://academic.oup.com/cid/advance-article/doi/10.1093/cid/ciy312/5000157. 10.1093/cid/ciy312 PMC6206101 29790925

[54] Folkehelseinstituttet (FHI) [Norwegian Institute of Public Health]. Covid-19, influensa og andre luftveisinfeksjoner: Rapport - uke 50 [Covid-19, influenza and other respiratory tract infections: report - week 50] [Internet]. 2022. Available from: https://www.fhi.no/contentassets/8a971e7b0a3c4a06bdbf381ab52e6157/vedlegg/2.-alle-ukerapporter-2022/ukerapport-uke-50-12.12--18.12.22.pdf.

[55] Jaksa A, Arena PJ, Chan KKW (2022). Transferability of real-world data across borders for regulatory and health technology assessment decision-making. Front Med.

